# Effect of ICU quality control indicators on VAP incidence rate and mortality: a retrospective study of 1267 hospitals in China

**DOI:** 10.1186/s13054-022-04285-6

**Published:** 2022-12-29

**Authors:** Xin Ding, Xudong Ma, Sifa Gao, Longxiang Su, Guangliang Shan, Yaoda Hu, Jieqing Chen, Dandan Ma, Feng Zhang, Wen Zhu, Guoqiang Sun, Xiaoyang Meng, Lian Ma, Xiang Zhou, Dawei Liu, Bin Du, China National Critical Care Quality Control Center Group, Xue Wang, Xiangdong Guan, Yan Kang, Bin Xiong, Bingyu Qin, Kejian Qian, Chunting Wang, Mingyan Zhao, Xiaochun Ma, Xiangyou Yu, Jiandong Lin, Aijun Pan, Haibo Qiu, Feng Shen, Shusheng Li, Yuhang Ai, Xiaohong Xie, Jing Yan, Weidong Wu, Meili Duan, Linjun Wan, Xiaojun Yang, Jian Liu, Hang Xu, Dongpo Jiang, Lei Xu, Zhuang Chen, Guoying Lin, Zhengping Yang, Zhenjie Hu

**Affiliations:** 1 Department of Critical Care Medicine, State Key Laboratory of Complex Severe and Rare Diseases, Peking Union Medical College and Chinese Academy of Medical Sciences, Peking Union Medical College Hospital, Beijing, 100730 China; 2 Department of Medical Administration, National Health Commission of the People’s Republic of China, 100044 Beijing, China; 3 Department of Epidemiology and Biostatistics, Institute of Basic Medicine Sciences, Chinese Academy of Medical Sciences (CAMS) & School of Basic Medicine, Peking Union Medical College, 100730 Beijing, China; 4 Information Center Department, State Key Laboratory of Complex Severe and Rare Diseases, Peking Union Medical College and Chinese Academy of Medical Sciences, Peking Union Medical College Hospital, Beijing, 100730 China; 5 Department of Medical ICU, State Key Laboratory of Complex Severe and Rare Diseases, Peking Union Medical College and Chinese Academy of Medical Sciences, Peking Union Medical College Hospital, Beijing, 100730 China

**Keywords:** Quality control, Ventilator-associated pneumonia (VAP), Incidence rate and mortality

## Abstract

**Purpose:**

To investigate the effects of ICU quality control indicators on the VAP incidence rate and mortality in China throughout 2019.

**Methods:**

This was a retrospective study. A total of 1267 ICUs from 30 provinces in mainland China were included. Data were collected using the National Clinical Improvement System Data that report ICU information. Ten related quality control indicators were analyzed, including 5 structural factors (patient-to-bed ratio, physician-to-bed ratio, nurse-to-bed ratio, patient-to-physician ratio, and patient-to-nurse ratio), 3 process factors (unplanned endotracheal extubation rate, reintubation rate within 48 h, and microbiology detection rate before antibiotic use), and 2 outcome factors (VAP incidence rate and mortality). The information on the most common infectious pathogens and the most commonly used antibiotics in ICU was also collected. The Poisson regression model was used to identify the impact of factors on the incidence rate and mortality of VAP.

**Results:**

The incidence rate of VAP in these hospitals in 2019 was 5.03 (2.38, 10.25) per 1000 ventilator days, and the mortality of VAP was 11.11 (0.32, 26.00) %. The most common causative pathogen was Acinetobacter baumannii (in 39.98% of hospitals), followed by Klebsiella pneumoniae (38.26%), Pseudomonas aeruginosa, and Escherichia coli. In 26.90% of hospitals, third-generation cephalosporin was the most used antibiotic, followed by carbapenem (24.22%), penicillin and beta-lactamase inhibitor combination (20.09%), cephalosporin with beta-lactamase inhibitor (17.93%). All the structural factors were significantly associated with VAP incidence rate, but not with the mortality, although the trend was inconsistent. Process factors including unplanned endotracheal extubation rate, reintubation rate in 48 h, and microbiology detection rate before antibiotic use were associated with higher VAP mortality, while unplanned endotracheal extubation rate and reintubation rate in 48 h were associated with higher VAP mortality. Furthermore, K. pneumoniae as the most common pathogen was associated with higher VAP mortality, and carbapenems as the most used antibiotics were associated with lower VAP mortality.

**Conclusion:**

This study highlights the association between the ICU quality control (QC) factors and VAP incidence rate and mortality. The process factors rather than the structural factors need to be further improved for the QC of VAP in the ICU.

**Supplementary Information:**

The online version contains supplementary material available at 10.1186/s13054-022-04285-6.

## Key message

- Eight ICU QC indicators including 5 structural factors (patient-to-bed ratio, physician-to-bed ratio, nurse-to-bed ratio, patient-to-physician ratio, patient-to-nurse ratio) and 3 process factors (unplanned endotracheal extubation rate, reintubation rate within 48 h, and microbiology detection rate before antibiotic use) were associated with VAP incidence rate.
- Only 2 process factors including unplanned endotracheal extubation rate and reintubation rate within 48 h were associated with high VAP mortality, while all the 5 structural factors were not.
- The process factors rather than the structural factors need to be further improved for the QC of VAP in ICU.

**Supplementary Information:**

The online version contains supplementary material available at 10.1186/s13054-022-04285-6.

## Introduction

As a life-saving intervention for a variety of critically ill patients, mechanical ventilation is vastly employed in the ICU all over the world. However, ventilator-associated pneumonia (VAP) is one of the most serious nosocomial infections in critically ill patients, which is associated with substantial morbidity and mortality, and considerable economical, psychological, and social costs to patients and families [1, 2]. VAP is preventable, and a combination of several core evidence-based elements, also known as VAP care bundle, is recommended. The VAP care bundle consists of 5 interventions: elevation of the head of bed between 30 and 45 degrees, daily sedative interruption and daily assessment of readiness to extubate, peptic ulcer prophylaxis, deep vein thrombosis prophylaxis, and daily oral care with chlorhexidine [3].

Although pieces of evidence suggest that, when adhered to VAP care bundle, the rate of VAP infections and the healthcare costs could be significantly reduced [4, 5], the VAP bundle compliance is not satisfactory [6, 7]. Education alone is not enough in VAP care bundle compliance [8], other methods such as checklists or real-time bundle adherence dashboards are also very important [9, 10]. And this phenomenon reminds us that the morbidity and the mortality of VAP are more related to the ability of implementing the bundles, which reflects the quality control level of ICU. Hence, there is an urgent need for the development and implementation of new strategies with the aim of improving care quality in ICUs.

The effect of quality improvement (QI) programs on clinical practice is gaining more and more attention in critical care medicine. A cluster randomized trial showed that multifaceted QI interventions improved the adoption of clinical practice in ICUs [11]. Marini et al. reported that multifaceted bundle interventions showed effective reduction in VAP rates in multidisciplinary ICUs [12]. An analysis of the National Clinical Improvement System Data in China highlighted the association between specific ICU structural factors and patients’ outcomes [13]. Therefore, the aim of this study was to investigate the ICU QI factors and VAP incidence rate and mortality and identify the association between these variables and patient outcomes.

## Methods

This was an observational database study in 2019, and the trial protocol was approved by the Central Institutional Review Board at Peking Union Medical College Hospital (NO. SK 1828), the approval included a waiver for the informed consent of patients and physicians. The data source was the National Clinical Improvement System((https://ncisdc.medidata.cn/login.jsp), collected by the China-National Critical Care Quality Control Center (China-NCCQC), which is the official national department that regulates ICU quality control in China. Peking Union Medical College Hospital was approved to establish the China-NCCQC in 2012 and initiated the quality improvement in Critical Care program in 2015. Analysis of the data was permitted by China-NCCQC.

### Study population and setting

The enrolled hospitals voluntarily participated and were selected by the China-NCCQC. The selection criteria were as follows. (1) The ICU had to have more than five beds. (2) The ICU met the requirements for equipment, construction, and management of ICUs in China. (3) The ICU had to have the ability to diagnose and treat the relevant medical diseases that were evaluated as quality control items (such as VAP). A trained data collector in each ICU was required to submit and report the quality control data via the Internet. The data in this study were collected between January 1, 2019, and December 31, 2019. Range checks were used to check for inconsistent or out-of-range data, prompting the user to correct or review data entries outside the pre-defined range.

### Variable and measurements

The QI factors of the ICU were evaluated according to the National Clinical Quality Control Indictors for Critical Care Medicine, which were officially recommended for the assessment of ICU performance by the National Health Commission of the People’s Republic of China in 2015 [14]. In this study, 10 related factors were analyzed, including 5 structural factors, 3 process factors, and 2 outcome factors. The structural factors included the ICU patient-to-bed ratio(calculated by the total number of ICU patients divided by the number of beds in the ICU), physician-to-bed ratio (calculated by the total number of ICU physicians divided by the number of beds in the ICU),nurse-to-bed ratio(calculated by the total number of ICU nurses divided by the number of beds in the ICU), patient-to-physician ratio (calculated by the total number of ICU patients divided by the number of ICU physicians), patient-to-nurse ratio (calculated by the total number of ICU patients divided by the number of nurse). The process factors included unplanned endotracheal extubation rate (calculated by the number of patients with unplanned endotracheal extubation divided by the number of patients with endotracheal extubation during the same period), reintubation rate within 48 h(calculated by the number of patients reintubated within 48 h after endotracheal extubation divided by the number of patients with endotracheal extubation during the same period), and microbiology detection rate before antibiotic use(calculated by the number of patients with microbiology detection before antibiotics divided by the number of patients who received antibiotics during the same period). The outcome factors included VAP incidence rate (‰) per 1000 ventilator days (calculated by the number of cases diagnosing VAP divided by the ventilator days during the same period) and VAP mortality rate (calculated by the number of patients who died of VAP divided by the number of patients diagnosing VAP). Moreover, the information on the most common pathogens and the commonly used antibiotics of VAP in the ICU was also collected.

### Data analysis

Descriptive analysis was made on the quality control indicators, including the structural factors, process factors, and outcome factors (VAP incidence rate and mortality). The continuous data were described by means of median (25th quantile, 75th quantile) and extreme value. As the incidence rate and mortality of VAP were relatively low, and the number of cases diagnosed and died of VAP showed discrete counting data at the hospital level, Poisson regression analysis was used to identify the impact of factors on the incidence rate and mortality of VAP. This process was realized through proc Genmod sentence. *P* < 0.05 was taken as statistically significant. All the analysis was completed by SAS 9.4 (SAS Institute, Inc., Cary, NC).

## Results

### Characteristics of the ICU QI factors

**Fig. 1:**
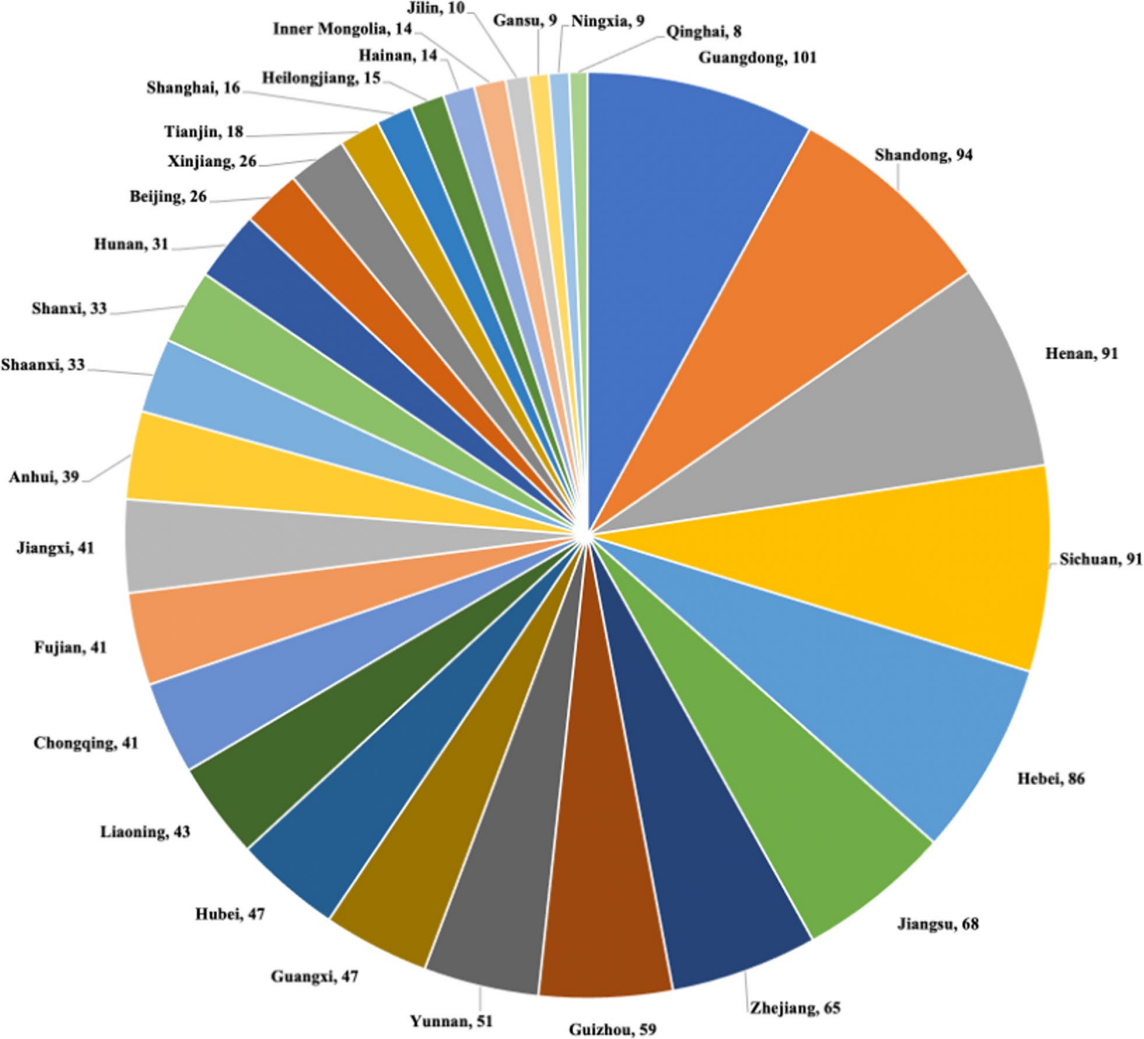
Proportions of hospitals from 30 provinces in this study

A total of 1267 hospitals and 109,1878 ICU patients from 30 provinces were included in this data analysis. The proportions of hospitals from each province are shown in Fig. 1. The average length of ICU stay was 6.35 (4.76, 8.83) days, the invasive mechanical ventilator days was 1548 (814, 3050), and the average ventilator days of ICU patients was 1.57 (2.65,4.31); other related data are listed in Additional file 1. The incidence rate of VAP in these hospitals was 5.03 (2.38, 10.25) per 1000 ventilator days, and the mortality of VAP was 11.11 (0.32, 26.00) %.

All the ICU factors’ characteristics are presented in Table 1. The median ICU patient-to-bed ratio was 37.64 (27.59, 51.55), the physician-to-bed ratio was 0.58 (0.44, 0.75), the nurse-to-bed ratio was 1.88 (1.50,2.31), the patient-to-physician ratio was 64.25 (45.63,90.25), and the patient-to-nurse ratio was 20.2 (14.4,28.62). The unplanned endotracheal extubation rate was 0.83 (0,2.14) %, the reintubation rate within 48 h was 1.76 (0.74,3.52) %, and the microbiology detection rate before antibiotic use was 92.86 (75.00,100) %. The most common causative pathogen was Acinetobacter baumannii (in 39.98% of hospitals), followed by Klebsiella pneumoniae (K. pneumoniae) (38.26%), Pseudomonas aeruginosa and Escherichia coli (Table 2a). In 26.90% of hospitals, third-generation cephalosporin was the commonly used antibiotics, followed by carbapenem (24.22%), penicillin and beta-lactamase inhibitor combination (20.09%), and cephalosporin with beta-lactamase inhibitor (17.93%) (Table 2b).

**Table 1:** Characteristics of the ICU quality improvement factors

Categories	Variables	N	Q1	Median	Q3	Extreme value
Structure factors	Patient-to-bed ratio	1267	27.59	37.64	51.55	3.19–252.20
	Physician-to-bed ratio	1267	0.44	0.58	0.75	0.12–3.19
	Nurse-to-bed ratio	1267	1.50	1.88	2.31	0.60–4.56
	Patient-to-physician ratio	1267	45.63	64.25	90.25	6.03–541.17
	Patient-to-nurse ratio	1267	14.4	20.2	28.62	2.28–99.91
Process factors	Unplanned endotracheal extubation rate (%)	1267	0	0.83	2.14	0–96.95
	Reintubation rate in 48 h (%)	1267	0.74	1.76	3.52	0–65.17
	Microbiology detection rate before antibiotic use (%)	1267	75.00	92.86	100	0.98–100
Outcome factors	VAP incidence rate (‰)	1267	2.38	5.03	10.25	0.00–344.44
	VAP mortality (%)	1267	0.32	11.11	26.00	0–100

**Table 2:**
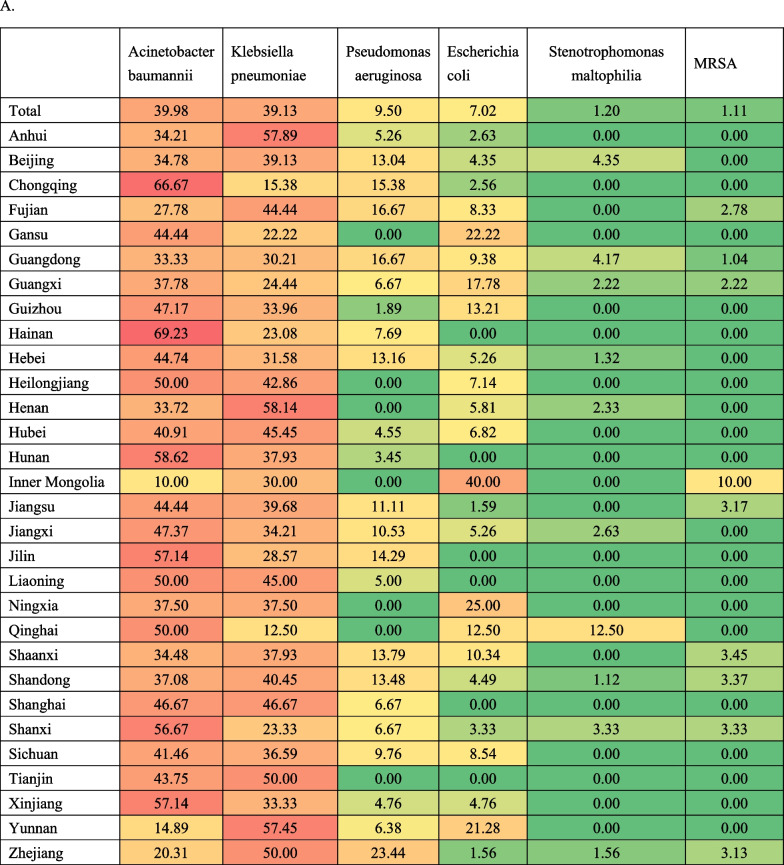

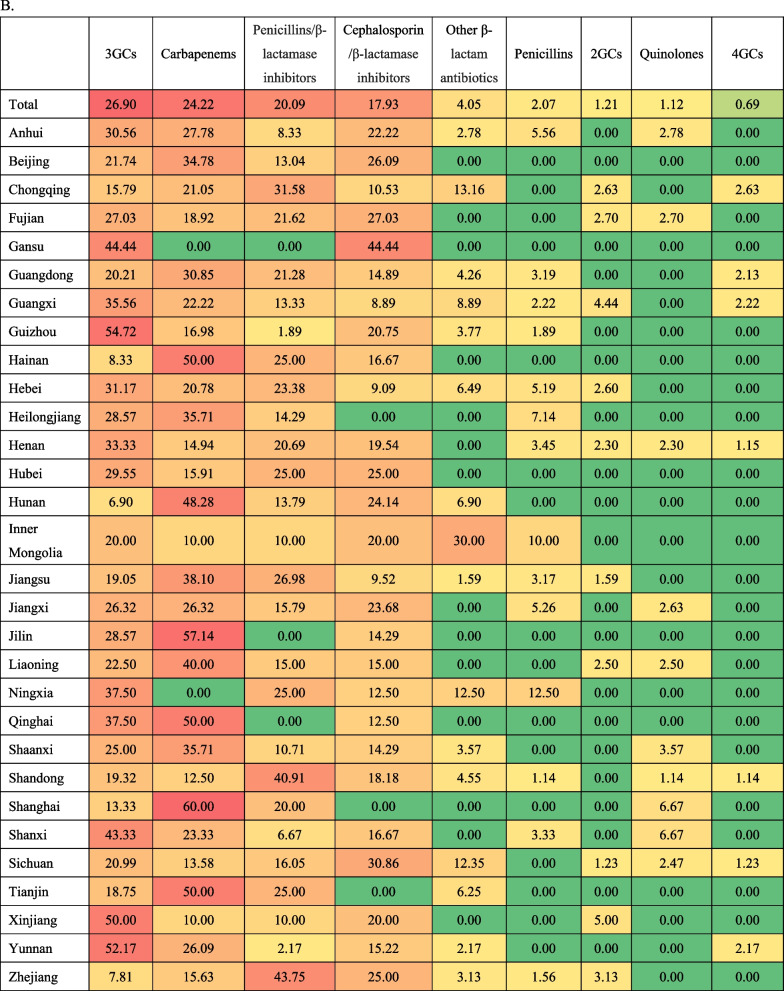
Distribution of infection pathogens and antibiotics of VAP among hospitals in 30 provinces of mainland China. **A**. Most common infection pathogens (proportion of hospitals, %). **B**. Most commonly used antibiotics (proportion of hospitals, %)

### Association between ICU QI factors and VAP mortality and incidence rate

**Fig. 2:**
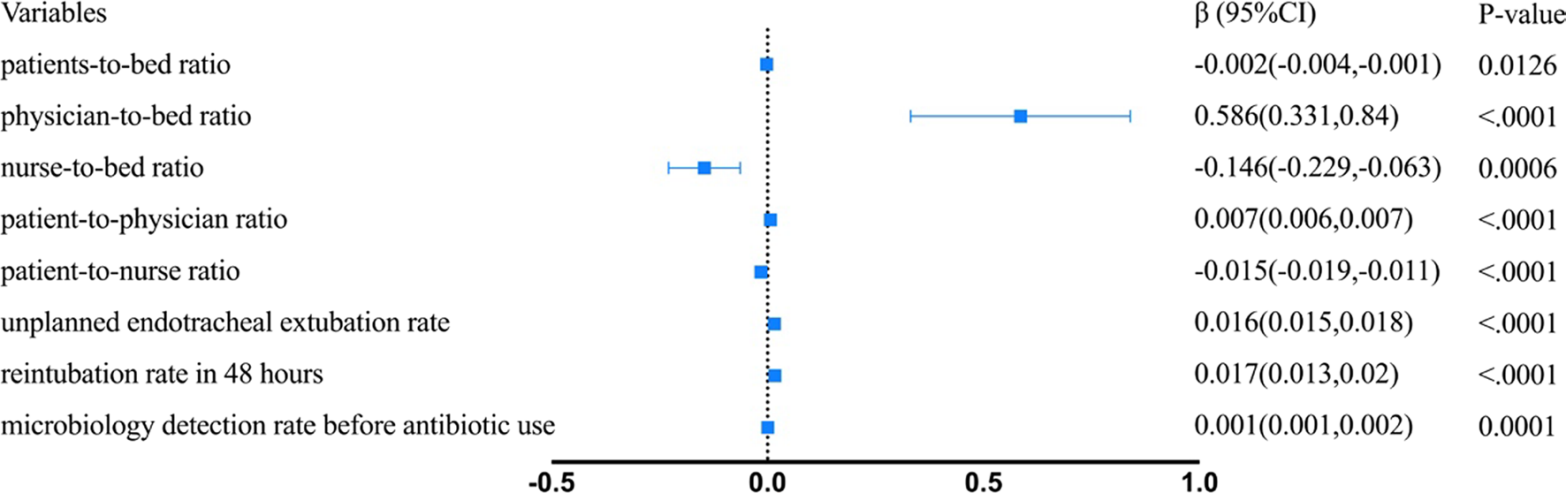
Adjusted effect of ICU structural factors and process factors on VAP incidence rate

The results of the Poisson regression analysis are shown in Fig. 2. Structural factors associated with lower ICU VAP incidence rate included patient-to-bed ratio (*β* = − 0.002 (− 0.004,− 0.001), *p* = 0.0126), nurse-to-bed ratio (*β* = − 0.146 (− 0.229,− 0.063), *p* = 0.0006), patient-to-nurse ratio (*β* = − 0.015 (− 0.019, − 0.011), *p* < 0.0001); and ICU VAP incidence rate was higher in hospitals with high physician-to-bed ratio (*β* = 0.586 (0.331,0.84), *p* < 0.0001) and patients-to-physician ratio (*β* = 007 (0.006,0.007), *p* < 0.0001). All the process factors were associated with higher ICU VAP incidence rate, including unplanned endotracheal extubation rate (*β* = 0.016 (0.015,0.018), *p* < 0.0001), reintubation rate in 48 h (*β* = 0.017 (0.013,0.02), *p* < 0.0001) and microbiology detection rate before antibiotic use (*β* = 0.001 (0.001,0.002), *p* < 0.0001).

**Fig. 3:**
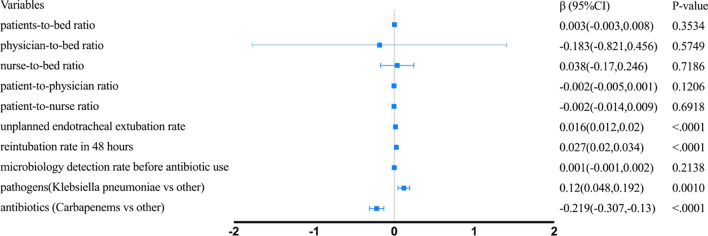
Adjusted effect of ICU structural factors and process factors on VAP mortality

All the structural factors were not significantly associated with VAP mortality. And process factors associated with higher VAP mortality included unplanned endotracheal extubation rate (*β* = 0.016 (0.012,0.02), *p* < 0.0001), reintubation rate in 48 h (*β* = 0.027 (0.02,0.034), *p* < 0.0001). K. pneumoniae as the most common pathogen was associated with higher VAP mortality (*β* = 0.12(0.048,0.192), *p* = 0.001), carbapenems as the most commonly used antibiotics were associated with lower VAP mortality (*β* = − 0.219(− 0.307, -0.13), *p* < 0.0001) (Fig. 3).

## Discussion

As one of the most common nosocomial infections in ICU, VAP constitutes a tremendous burden for the critically ill patients, and the prevention and management of VAP were paid close attention. Although the strategies of diagnosis, prevention, and management have been recommended in several guidelines [15, 16], and effective implementation of VAP care bundles was associated with superior clinical and economic outcomes [5], factors affecting the effectiveness of these strategies were rarely studied. To our knowledge, this is the first national report which analyzes the influencing factors of VAP mortality and incidence rate from the perspective of ICU quality improvement. And we found that not only ICU structural factors such as patient-to-bed ratio, physician-to-bed ratio, nurse-to-bed ratio, patient-to-physician ratio, and patient-to-nurse ratio were associated with VAP incidence rate, but also the process factors including unplanned endotracheal extubation rate and reintubation rate in 48 h and microbiology detection rate before antibiotic use. However, all the structural factors were not significantly associated with the ICU mortality, and only unplanned endotracheal extubation rate, reintubation rate in 48 h were associated with higher VAP mortality. Furthermore, VAP mortality was higher in hospitals with K. pneumoniae as the most common infection pathogens, and lower in hospitals with carbapenems as the most commonly used antibiotics.

Although critical care medicine in mainland China had made great progress, there was still a large gap with developed countries in the number of ICU beds and capacity, equipment, clinician staffing, ICU technicians, and so on. The physician-to-bed ratio and nurse-to-bed ratio were recommended in the Guidelines of Construction and Management of Critical Care Medicine in China, but many hospitals didn’t meet this recommendation [17]. While the number of hospitals and hospital volumes is gradually increasing during the past few years in China, the human resources were insufficient to keep pace with the increase in the number of ICU beds [14]. Traditionally, adequate human resource allocation is one of the core elements needed to ensure the quality of medical care [18], as ICU patients are highly dependent on nursing care, a shortage of nursing staff could be associated with insufficient supervision and recognition of state changes of the patients. Saker et al.’s study found that a high nurse-to-patient ratio was independently associated with a reduced risk of in-hospital mortality [19], and Li et al. found a similar result [13]. In our study, a high nurse-to-bed ratio was also associated with a lower VAP incidence rate, but other factors such as patient-to-nurse ratio and physician-to-bed ratio showed a different trend. More interestingly, all the structural factors were not associated with VAP mortality. To our surprise, our study found that a higher physician-to-bed ratio was associated with a higher VAP incidence rate, and it may be partly explained by the special situation of the development of ICU in China. Intensive care medicine was officially approved as an independent discipline in China until 2008, after the severe Wenchuan earthquake. In this context, while most nurses are engaged in ICU after graduation, a considerable number of ICU physicians are from other professions. Compared with the nurses, the concept and behavior of physicians in the prevention of nosocomial infection are still relatively weak, despite several national continuing medical educations. We believe that, with the continuous improvement in ICU education system, this situation will improve eventually. On the other hand, we have to realize that the number of patients and medical staff is in constant dynamic change in clinical practice, and the structural factors such as patient-to-physician and patient-to-nurse ratio may not accurately reflect the clinical workload. However, these results reminded us that, at least for VAP, the quantity of the medical staff was not the only decisive factor, and simply focusing on the quantity of the medical staff was not enough for improving the incidence rate and mortality of VAP. A performance assessment of medical professionals in the prevention of VAP showed that only 52.6% had satisfactory performance [20], and conditions were even worse in low-income countries such as Tanzania [7]. Factors that could reflect the medical performance such as process factors should be noticed.

Different from the structure factors, process factors were not only associated with the VAP incidence rate, but also the VAP mortality. We found the both the unplanned endotracheal extubation rate and reintubation rate in 48 h were associated with a higher incidence rate and mortality of VAP in ICU patients, but the microbiology detection rate before antibiotic use was only associated with a higher VAP incidence rate. Unplanned endotracheal extubation is a major complication of translaryngeal intubation and sometimes can cause reintubation [21], while other causes of reintubation could be recurrent pneumonia and so on. Epstein et al. reported that 56% of patients required reintubation after unplanned extubation, and it resulted in prolonged MV, longer ICU, and hospital stay [21]. A systemic review in pediatric ICU found that risk factors associated with unplanned extubation were age, inadequate tube fixation, agitation, copious secretions, performance of patients’ procedures, and nursing workload [22]. A study that enrolled 17 unplanned extubation in 15 patients reported that patients who suffered an episode of unplanned extubation had an increased risk for VAP [23]. Another prospective multicenter study showed that unplanned extubation after weaning increased the risk of nosocomial pneumonia [24]. Results obtained for 16 studies in a meta-analysis found that reintubation was a risk factor for the development of VAP (26), and meta-analyses of 195 studies in mainland China also found that reintubation was significantly associated with the occurrence of VAP [25]. Improvement in quality components was effective in reducing unplanned extubation [22]. There were few studies exploring the relationship between these two factors and VAP mortality, some studies showed that unplanned extubation didn’t influence the ICU or hospital mortality [21, 24]. Unplanned endotracheal extubation rate and reintubation rate in 48 h were factors that reflected the ability of airway management from different aspects, their significant association with VAP incidence rate and mortality further highlights the importance of process factors in the ICU QI program.

The information on leading infectious pathogens of VAP and the most commonly used antibiotics were also collected. Gram-negative organism was the main causative agents of VAP [26], pathogens such as Acinetobacter baumannii, Pseudomonas aeruginosa, and Klebsiella pneumoniae were frequently associated with VAP in ICU [27], with Acinetobacter baumannii was often the most common pathogens [28–30]. However, recent studies have shown an increase in the prevalence of Klebsiella pneumoniae [31–34], especially in the context of the prevalence of carbapenem resistance Klebsiella pneumoniae. The data of CHINET in 2018 showed that Klebsiella pneumoniae was the most common pathogen isolated from the lower respiratory tract, and the resistance rates of Klebsiella pneumoniae to imipenem and meropenem were increased to 25% and 26.3% respiratory from 3.0 to 2.9% in 2005 [35]. Yi et al. found that 44.0% of Klebsiella strains were carbapenem resistant, and VAP non-survivors had a higher prevalence of CRKP than VAP survivors [34]. Xu et al. reported that patients infected with CRKP have high mortality than those infected with CSKP, especially in association with ICU admission. The data in our study were similar, Acinetobacter baumannii was still the most common pathogen of VAP in 39.98% of hospitals, but only a little higher than Klebsiella pneumoniae (39.13%). Considering the increasing prevalence of Klebsiella pneumoniae recently, especially the carbapenem-resistance Klebsiella pneumoniae, we wonder whether these conditions could influence the VAP mortality. In this study, the hospitals with K. pneumoniae as the most common pathogen of VAP had significantly high mortality than those without, and we hypothesized that increasing resistance to carbapenem may play a role, which needed further investigations.

The choice of antibiotics also influenced the VAP mortality. Our data showed that 24.22% of hospitals choose carbapenems as the first choice to treat VAP, only slightly lower than the third-generation cephalosporin (26.90%). In hospitals with carbapenems as the most commonly used antibiotics, the VAP mortality rate was lower. This result should be explained with caution, as the study object here was hospital but not the patients. In Arthur et al.’s study, four studies compared carbapenems with other antibiotics in VAP treatment [36]. The only study to report all-cause mortality by Freire et al. showed no significant difference [37]. The other three studies showed that treatment with carbapenems had significantly higher clinical cure when compared to non-carbapenems including tigecycline, levofloxacin, and piperacillin–tazobactam [38]. A recent meta-analysis found that carbapenem-based empiric regimens were associated with lower mortality rates compared with non-carbapenems, largely driven by trials of VAP. But there was a trend toward increasing resistance associated with carbapenems [39]. As we know, the selection pressure of carbapenem antibiotics on Enterobacteriaceae was beneficial to the screening of carbapenem-producing strains, which may lead to the mass production of CRE strains [40]. Therefore, although results told us, carbapenem antibiotics were still a powerful means to treat VAP, they should still be used with caution.

There were a few limitations of this study. First, this was an observational study and was prone to selection bias. Causal relationships can’t be drawn due to the cross-sectional nature of the study. Second, the data from this study were from the National Clinical Improvement System in 2019, and the information on VAP was only a small part of the database, and detailed information associated with VAP such as the oral hygiene, subglottic secretions management was not included. Further studies were needed to identify the direct influencing factors of the incidence of VAP. Third, the association between the causative pathogens or antibiotics with VAP mortality should be explained with caution. On the one hand, they were descriptive results of the overall situation of the hospitals but not the patients; on the other hand, important information such as the antibiotics resistance information of the local hospital was not available. Despite these limitations, the results of this study are highly meaningful that they underscore the ICU process factors but not the ICU structural factors which were more important and may provide more evidence for the critical care developments in China.

## Conclusion

In conclusion, ICU structural factors including patient-to-bed ratio, physician-to-bed ratio, nurse-to-bed ratio, patient-to-physician ratio, and patient-to-nurse ratio were associated with VAP incidence rate, but not with VAP mortality. Two of the three process factors including unplanned endotracheal extubation rate and reintubation rate within 48 h were associated with VAP incidence rate and mortality, while microbiology detection rate before antibiotic use was only associated with VAP incidence rate. The process factors rather than the structural factors need to be further improved for the QC of VAP in ICU.

## Supplementary Information

**Additional file 1: Table S1**. Characteristics of the other relative factors in the study.

## Data Availability

The datasets analyzed during the current study are available from the corresponding author upon reasonable request.
