# Supplementary material for: Effect of ICU quality control indicators on VAP incidence rate and mortality: a retrospective study of 1267 hospitals in China
Source: Crit Care. 2022 Dec 29;26:405. doi: 10.1186/s13054-022-04285-6 (PMC9798551; doi:10.1186/s13054-022-04285-6)
Supplement: Supplementary file 1 — Additional file 1: Table S1. Characteristics of the other relative factors in the study. [file 13054_2022_4285_MOESM1_ESM.docx]

Table 1 Characteristics of the other relative factors in the study

| Variables | Summation | Q1 | Median | Q3 |
| --- | --- | --- | --- | --- |
| No. of patients admitted to ICU | 1091878 | 361 | 568 | 946 |
| No. of ICU patients bed days | 7837407 | 2267 | 3743 | 6563 |
| Cases of VAP in ICU | 21366 | 3 | 9 | 18 |
| No. of ventilator days in ICU | 3350972 | 814 | 1548 | 3050 |
| No. of patients died with VAP | 3244 | 0 | 1 | 3 |
| Average length of ICU stay (days) |  | 4.76 | 6.35 | 8.83 |
| Average length of stay (days) |  | 7.25 | 8.25 | 9.63 |
| Average ventilator days of ICU patients |  | 1.57 | 2.65 | 4.31 |
